# Associations Between Reward and Future-Related Orientations and General and Specific Mental Health Issues in Adolescence

**DOI:** 10.1007/s10802-023-01136-y

**Published:** 2023-10-07

**Authors:** Yi Yang, Xinxin Zhu, Bonnie Auyeung, Ingrid Obsuth, Aja Murray

**Affiliations:** 1 Department of Psychology, University of Edinburgh, Edinburgh, UK; 2 Clinical Psychology Department, University of Edinburgh, Edinburgh, UK

**Keywords:** Delay discounting, Transdiagnostic, Bi-factor model, Future orientation, Risk-taking, Cross-informant

## Abstract

Adolescence is characterised by a peak in sensation seeking accompanied by gradually developing self-control skills. Adolescents typically show steeper delay discounting performance than other age groups; a feature that is transdiagnostically related to a variety of mental health disorders. However, delay discounting performance is not a singular mental process but involves both risk/reward and future orientation elements, usually operationalised as probability/risk and time discounting tasks, respectively. To clarify the specific relations between the risk/reward and future orientation elements of delay discounting and different types of mental health problems, two bi-factor models and a series of structural equation models (SEMs) were fitted to multi-informant (parent and adolescent self-reported) mental health data from a large UK study. A transdiagnostic promotive role of future orientation was found using bi-factor modelling to separate general and dimension-specific mental health variation; however, this was limited to parent reports. In addition, future orientation was negatively associated with conduct problems and ADHD symptoms, but positively associated with emotional problems. Risk aversion was negatively associated with conduct problems, but positively associated with emotional and peer problems. The findings highlight that risk/reward and future orientation elements of delay discounting play partly distinct roles in different mental health problems and can serve both promotive and risk roles during adolescence. Findings also illuminate which elements of delay discounting should be intervention targets for different mental health concerns.

**Supplementary Information:**

The online version contains supplementary material available at 10.1007/s10802-023-01136-y.

## Introduction

Adolescence is a critical developmental period for mental health problems, with symptoms commonly showing their first onset during this time (Kessler et al., 2007; Paus et al., 2008; Solmi et al., 2022). A considerable body of research has shown that different mental symptoms in adolescence are correlated across different domains (Allegrini et al., 2020; Clark et al., 2021; Murray et al., 2016). This has motivated research identifying potential ‘transdiagnostic’ risk and promotive/protective factors (e.g., Dvorsky & Langberg, 2016; Farrell et al., 2010) that impact mental health symptoms across multiple domains. These transdiagnostic factors are considered promising targets for prevention and intervention that can be leveraged to more efficiently mitigate or improve, respectively, mental health problems across multiple different domains simultaneously. Delay discounting tendencies have previously been identified as correlated with diverse symptoms of mental health disorders (Amlung et al., 2019), making delay discounting processes a promising set of candidate transdiagnostic mental health factors. However, at the same time, delay discounting performance has been found to activate multiple neural system processes (McClure et al., 2004) and these may play different roles in relation to different mental health problems. The purpose of the present study is, therefore, to explore the extent to which (i) elements of delay discounting act as transdiagnostic predictors of mental health problems and (ii) which specific dimensions of mental health problems are uniquely predicted by different elements of delay discounting measures after accounting for the tendency for symptoms of mental health problems to co-occur.

Delay discounting is commonly used as an index of impulsive decision-making (Moreira & Barbosa, 2019; Rubia et al., 2009; Teuscher & Mitchell, 2011). Its tasks typically require participants to choose between a smaller more immediate reward and a larger later reward, with both delays and rewards varying gradually across trials. For example, the task may vary the amount of reward over several trials for one week, one month, one year and so on. Researchers then usually evaluate how much of the long-term reward participants are willing to wait for or invest in for each of the delays (Levin et al., 2018; Nguyen et al., 2018; Rung & Madden, 2018), to determine their “indifference points” (Rodzon et al., 2011). The indifference points for delays are then plotted, with the curve-fitting analysis yielding a k-value, which provides a quantitative index of the steepness of the discounting curve (Mazur, 1987; Rodzon et al., 2011) and impulsivity of decision-making (Rung & Madden, 2018).

A recent meta-analysis by Amlung and colleagues (2019) examined the links between delay discounting and mental health problems. Their review included 57 effect sizes from 43 studies across 8 diagnostic categories and found that steeper delay discounting is transdiagnostically associated with a wide range of mental health conditions, with steeper discounting related to greater mental health issue risk. By comparing individuals with a psychiatric disorder with controls, they found reliable aggregate effect sizes for the delay discounting k-value differences for a range of conditions, including major depressive disorder with Hedges *g* = 0.37 for 7 studies, schizophrenia with Hedges *g* = 0.46 for 12 studies, borderline personality disorder with Hedges *g* = 0.60 for 8 studies, bipolar disorder with Hedges *g* = 0.68 for 4 studies, bulimia nervosa with Hedges *g* = 0.41 for 4 studies, and binge-eating disorder with Hedges *g* = 0.34 for 7 studies. This adds to previous meta-analytic evidence that attention-deficit hyperactivity disorder (Doidge et al., 2018; Marx et al., 2021) and addictive disorders (Amlung et al., 2017) are also associated with steeper delay discounting. Taken together, these studies point to small to medium magnitude associations between delay discounting and a range of mental health problems.

However, these meta-analyses only considered pairwise associations between delay discounting and different mental health problems. Given that symptoms of mental health problems tend to sequentially and concurrently co-occur in adolescence, it is valuable to examine the links between delay discounting and mental health symptoms in a model that can appropriately capture the links between symptoms in different domains. A popular method for examining the relations between a candidate transdiagnostic risk orpromotive factor and mental health outcomes is the use of a bi-factor structural equation model (e.g., Noordhof et al., 2015; Smith et al., 2020). The bi-factor model is a type of confirmatory factor analysis model, which assumes that the general and subfactors are all orthogonal and facilitates a separation of variation that is shared among all mental health symptoms and variation that is unique to specific mental health domains (Gibbons & Hedeker, 1992; Gomez et al., 2019; Reise, 2012). Here, a general factor of psychopathology (sometimes termed the ‘p-factor’) accounts for the covariance of psychopathology symptoms or diagnosis across all items, while the subfactors in the bi-factor model capture the covariance of specific clusters of items after accounting for the p-factor (Reise, 2012). Previous studies using this approach have suggested that a p-factor dimension can parsimoniously model the existence and persistence of co-occurring mental health problems, as well as the emerging psychopathological structure of psychiatric disorders (Caspi et al., 2014).

Further, p-factor analyses have facilitated the analyses of shared risk factors and biomarkers, and common responses to the same therapies (Caspi & Moffitt, 2018). In principle this means that targeting this shared risk could potentially intervene in a wide range of mental health problems at once, providing an efficient prevention and intervention target. On the basis of this, if a risk or promotive factor predicts the p-factor, it conceptually suggests the factor transdiagnostically predicts shared risk or vulnerability across disorders and potentially accounts for the tendency for different mental health symptoms to co-occur. Its association with some subfactor(s) beyond the p-factor, on the other hand, means in addition to influencing that specific mental issue via affecting one’s general mental health or psychopathology (e.g., promoting resilience), the transdiagnostic factor could also have a direct impact on that mental issue via its unique pathway (e.g., treating psychological symptoms) (Caspi et al., 2014; Wright & Masten, 2014). This also helps address the issue that – due to widespread correlations between different mental health problems – the relations between a risk or promotive factor and a specific domain of mental health problems may be confounded to the presence of co-occurring issues. However, there remain some contradictory findings on the extent to which a p-factor is supported using bi-factor model, with some studies finding evidence for a strong p-factor with loadings across a wide range of symptoms (e.g., Caspi et al., 2014; 2018) and some studies failing to find such support (Eid, 2020; Eid et al., 2017). As such, an important first step in examining transdiagnostic predictors of mental health problems using bi-factor modelling is to examine and interpret the extent to which there is truly symptom-general variation captured by a p-factor in a given dataset.

To our knowledge, to date, only two studies have examined the association between delay discounting and mental health problems using a bi-factor modelling approach. One bi-factor study utilised a US-based sample of 602 participants with an average age of 22.63 and did not find an association between delay discounting and alcohol use disorder, ADHD or the shared variance among them (Oddo et al., 2021). In contrast, in another bi-factor study that utilised a sample of 2,144 participants with an average age of 14.39 across eight European sites, the relations between conduct disorder, ADHD, oppositional-defiant disorder (ODD), obsessive-compulsive disorder, eating disorders, substance use, anxiety, depression, phobias, and other emotional symptoms were well characterised by a revised version of bi-factor model with allowing specific factors to covary. Steeper delay discounting was associated with the general factor (p-factor) in this model, suggesting that it acts as a transdiagnostic risk factor (Castellanos-Ryan et al., 2016). These inconsistent findings point to the necessity of clarifying the association between delay discounting and general and specific variance in mental health.

When considering delay discounting as a potential transdiagnostic factor in mental health difficulties, it is also important to address the fact that delay discounting engages multiple neural system processes (McClure et al., 2004). Indeed, the classical delay discounting task involves both varying delays (future orientation) and rewards (risk/reward) at the same time. The neurological finding underpins the theory that delay discounting involves the competition between reward-seeking and future orientation processes, with reward-seeking predominance leading to impulsive/risk decision-making and future orientation predominance leading to delay preference (Steinberg et al., 2009). Accordingly, a recent experimental examination by Jiang and Dai (2021) found causal pathways from time and risk perceptions to delay discounting by manipulating risk and time perception respectively in two experiments. Supporting the importance of this distinction, a meta-analysis by Johnson et al. (2020) on 26 studies (totalling 32 effect sizes), which also included discounting tasks on reward probability/risk (probability/risk discounting) that particularly captured the risk-taking preference facet of delay discounting, found only a small to moderate association between risk-taking preferences and delay preferences, suggesting that time-waiting discounting (e.g., $50 today vs. $100 in 3 months) and risk-taking discounting (e.g., a certain $50 vs. a 50% probability of $100) may reflect distinct pathways. Adolescence is characterised by a peak in sensation-seeking desire but only gradually developing self-control skills (Icenogle & Cauffman, 2021; Steinberg, 2008), making it a time of special developmental significance for risk-related decision-making. Clarifying the specific relations between the two pathways of delay discounting and different types of mental health issues during this period could, therefore, be especially informative for the prevention of mental health issues. This could also illuminate more specific and developmentally tailored clinical intervention methods.

A final consideration is that it is well known that young people may exhibit different behaviours in different contexts and/or with different informants, giving each informant a partially unique perspective. A multi-informant approach has thus been considered the best practice for examining the behaviours of children and adolescents in mental health research. The review studies by De Los Reyes et al. (2013;, 2015) analysed existing cross-informant research in clinical science, such as developmental psychopathology, controlled trials research, and personality disorders assessments, and found support for interpreting multiple informants’ reports as each providing a valid but unique perspective. Their reviews defined two approaches: while a convergent operation assumes human behaviour consistency across settings and time and treats any discrepancy as measurement error, a divergent operation treats discrepancies as human behavioural variation patterns responding to environment and time differences (De Los Reyes et al., 2013; De Los Reyes et al., 2013). Importantly, despite the fact that the converging operations approach is widely applied in the existing literature, it raises challenges in interpreting research findings, since it is widely recognized that people behave differently in relation to environmental contingencies as a result of immediate reinforcement or punishment (Skinner, 1953). Achieving convergence between reports may meet researchers’ expectations but sacrifices valuable information about behaviours across settings and time observed by different informants. Therefore, going beyond converging operations to interpret variation patterns from multiple informants’ reports is important and deserves more research.

The present study therefore adopted this divergent approach. To rule out the possibility of cross-informant discrepancies attributed to methodological factors, multigroup confirmatory factor analysis is widely used to test measurement invariance across informants. If at least metric invariance holds (factor loadings of a CFA model are shown to be equal across informants), then this suggests that the concepts have the same meaning to different informants. This suggests that discrepancies do not reflect different understandings of mental health concepts, interpretations of behaviours, or response tendencies to items, and facilitates a descriptive comparison of patterns of associations in the different informant reports (e.g., Murray et al., 2021).

### The Present Study

To help clarify the extent to which different elements of delay discounting are transdiagnostic versus disorder-specific risk or promotive factors in mental health problems, the present study fitted separate bi-factor models to parent and youth mental health data from a large UK-based study, and correlated the resulting general and specific dimensions of mental health problems with both time discounting and risk discounting.

Given that existing research has converged to support the idea that mental health can be divided into general and specific variances and that future orientation is associated with a wide range of mental health problems (e.g., Baird et al., 2021; Kooij et al., 2018), our research hypotheses were as follows:

#### Hypothesis 1

Parent-reported adolescent mental health problems can be captured by a general factor using a bi-factor modelling approach;

#### Hypothesis 2

Adolescent self-reported mental health problems can be captured by a general factor using a bi-factor modelling approach;

#### Hypothesis 3

The future orientation component of delay discounting will transdiagnostically predict the p-factor operationalised in these bi-factor models;

All other analyses are exploratory.

## Methods

### Participants

Data utilised in the current study were from sweep 7 of the existing Millennium Cohort Study (MCS). Given that delay discounting tasks were only assessed at this sweep which is also the latest sweep with available data, a longitudinal analysis was not possible. Participants in sweep 7 turned age 17 between January 2018 to March 2019 when the data were collected. Their parents also provided self-report information as well as information regarding the participants and their families at this point (N = 10,834, Female = 50.1%). All participants provided informed consent. Ethical approval for the main stage at sweep 7 was obtained on 18th October 2017 from the National Research Ethics Service (NRES) Research Ethics Committee (REC) North East – York (REC ref: 17/NE/0341; “MCS7_Technical_Report,” 2020). MCS is a UK-based nationally representative longitudinal cohort study using a clustered, stratified sampling procedure on children born in the UK between September 2000 and January 2002. MCS has been following the development of more than 18,000 individuals and their families from 9 months old with subsequent waves taking place at ages 3, 5, 7, 11, 14, and 17, and the data collection is still ongoing. For further information, see MCS sampling procedures, cohort profiles and documentation (Connelly & Platt, 2014; Joshi & Fitzsimons, 2016; Plewis, 2007). The full documentation and access are available at: https://ukdataservice.ac.uk.

### Measures

**Strengths and Difficulties Questionnaire (SDQ)**: The SDQ (Goodman, 1997) was completed by both young people and their parents. It is a brief behavioural screening questionnaire suitable for individuals ages 2–17 and one of the most widely used behavioural screening instruments. It contains 25 items in 5 subscales measuring emotional problems (5 items), conduct problems (5 items), hyperactivity/inattention (5 items), peer problems (5 items), and prosocial behaviour (5 items). The present study only used the 20 difficulty items: on emotional problems, conduct problems, hyperactivity/inattention (ADHD), and peer problems. Each item was rated on a 3-point Likert Scale, with 0 = not true, 1 = somewhat true, and 2 = certainly true. Higher scores represent greater levels of each symptom. The reversed items were rescored reversely before analysis. According to a systematic review of the evidence on the psychometric properties of this measure, the structural, discriminative and convergent validity have all been supported, with some minor caveats (Kersten et al., 2016). Previous work also found favourable psychometric properties of the SDQ specifically in the current sample, including longitudinal, gender, and informant invariance in adolescents (Murray et al., 2021, 2022).

#### ED50 Delay Discounting Tasks

The MCS study adapted the ED50 method for delay discounting from Koffarnus and Bickel (2014), which is based on the same testing effect but is more concise than the traditional version, by using 10 trials with only one delay/probability instead of several levels of delays/probabilities. This is illustrated in Figures S1 and S2 in supplementary materials. The future orientation aspect of delay discounting was operationalised as the “Time Preference” (see Figure S1 in supplementary materials) discounting task in the MCS study. By fixing two delays and varying rewards, this particularly quantifies the psychological monetary equivalence for the time preference (future orientation) between “2 months” and “4 months” over a series of monetary choices (e.g., “£50 in 2 months or £80 in 4 months”), with higher k values indicating higher delayed-time preference/future orientation. The risk/reward preference of delay discounting was operationalised as the “Risk” (see Figure S2 in supplementary materials) discounting task. By fixing two probabilities and varying rewards, this quantifies the psychological monetary equivalence for risk aversion (or certainty preference) between 50% certainty and 100% certainty over a series of monetary choices (e.g., “a 50–50 chance of £240 or £72 for certain”), with higher k values indicating higher risk aversion (or certainty preference). According to the formula for delay discounting V = A/(1 + kD), 1/k is equal to the delay (D) where the current value of a reinforcer (V) is half of its nominal amount (A). This ED50 delay, also called the Effective Delay 50%, is therefore more intuitively understandable as a measure of discount rate and is easily converted from or to a k value. This relationship also suggests that an assessment to directly measure an individual’s ED50 value for a commodity would be an effective measure of that individual’s discount rate for that commodity. For more information regarding the ED50, see Koffarnus and Bickel (2014).

To aid interpretation, the “Time Preference” discounting task scores (the higher k value, the longer time delayed) were labelled future orientation, and the “Risk” discounting task scores (the higher k value, the lower risk-taking, or higher risk aversion, or higher certainty preference) were labelled risk aversion for the present study.

### Statistical Analysis

Excel was used to calculate the ED50 and k_ED50_ values of the delay discounting tasks. SPSS was used for transforming the ED50 and k_ED50_ values to standardized scores. Pearson correlations among all the factors, and the paired sample t-test between each of the participants’ self-reported and parent-reported SDQ each subfactors were calculated. R software was employed to conduct the statistical analysis. All the data were treated as categorical and missing data were dealt with using “pairwise” deletion in the lavaan package (Rosseel, 2012) of R with DWLS as estimator.

**Confirmatory factor analysis (CFA**): A conventional cut-off point for salient item loadings of ≥|0.30| was adopted. Model fit was assessed using the following indices: the comparative fit index (CFI), Tucker–Lewis index (TLI), the root-mean-square error of approximation (RMSEA) and the Standardized Root Mean Squared Residual (SRMR). Model fits cut-offs used to judge good fit were: > 0.90 for incremental fit indexes (CFI and TLI), < 0.08 for RMSEA, and < 0.08 for SRMR (Bentler & Bonett, 1980; Browne & Cudeck, 1992; Hu & Bentler, 1999).

#### Bi-factor Analysis

Bi-factor models were examined by specifying a general p-factor which contains all the items, as well as four subfactors (emotional problems, conduct problems, ADHD, peer problems), and fixing the correlations among the general p-factor, emotional problems, conduct problems, ADHD, and peer problems to zero. Then, the standardized k_ED50_ values for future orientation and risk aversion were used to covary with both the p-factor and the four subfactors (emotional problems, conduct problems, ADHD, and peer problems). This was repeated for the self- and parent-reported SDQ items, which resulted in two separate bi-factor models. A conventional cut-off point for salient item loadings of ≥|0.30| for a majority of general factor items loadings (to ensure the factor was truly general) and specific factor loadings, in conjunction with good model fits was used as the standard for an acceptable bi-factor model.

#### Multiple Group Confirmatory Factor Analysis (MG-CFA)

To ensure a meaningful descriptive comparison and interpretation of the two separate bi-factor models from different informants in a divergent approach, multiple group confirmatory factor analysis was conducted to empirically examine whether the bifactor model captured equivalent constructs across informants. MG-CFA was used with a series of model comparisons defined with more and more stringent equality constraints. Within the CFA and bi-factor and bi-factor SEM frameworks, baseline configural invariance is judged to hold if the model achieves the conventional cut-offs for good model fits, such as CFI > 0.90 and RMSEA < 0.08 (Pendergast et al., 2017). There is a lack of agreement among previous studies on the thresholds of changes of model fits between different levels of invariance models to determine measurement equivalence across groups (see a summary by Svetina et al., 2020); however, for the purposes of the present study, it was judged reasonable to use the widely used cut-off values proposed by Chen (2007) based on a comprehensive simulation study. Since we only focused on the correlations between delay discounting and mental health variables, a weak/metric level of invariance is required to support descriptive comparisons across informants. Metric invariance would be supported by a lack of substantial decrease of CFI and increase of RMSEA and SRMR than the configural invariance model (ΔCFI ≥ − 0.01, ΔRMSEA ≤ 0.015, ΔSRMR ≤ 0.03; Chen, 2007), which means the magnitudes of the relationships between items and p-factor/subfactors are equivalent across groups, signalling no significant difference in understandings of the measurement constructs.

## Results

The means, standard divisions, and raw correlations for all the variables are provided in Tables 1 and 2. All the youth self-reported and parent-reported SDQ symptom variables were positively and significantly correlated. Paired samples t-tests (Table 3) suggested that all four SDQ subfactors from self-reports were significantly higher than the corresponding parent reports. As shown in Table 2, future orientation was positively related to self-reported emotional problems while negatively related to other SDQ problems for both informants; however, its correlations with adolescent self-reported peer problems and ADHD problems, and with parent-reported emotional problems were not significant. Risk aversion was negatively related with adolescent self-reported conduct problems while positively related with other SDQ problems cross-informants; but its relations with adolescent self-reported ADHD symptoms and parent-reported conduct problems were not significant. Future orientation and risk aversion were negatively and significantly related.

**Table 1:** Descriptive Statistics

	Mean	Std. Deviation	N
kcertain	0.01	0.01	6280
ktime	0.01	0.00	6648
CES	0.70	0.49	9655
CPR	0.43	0.34	9684
CCP	0.34	0.30	9676
CADHD	0.79	0.46	9677
PES	0.41	0.45	7462
PPR	0.35	0.36	7464
PCP	0.23	0.29	7490
PADHD	0.49	0.45	7520

Note: kcertain = k Values for risk aversion of Delay Discounting; ktime = k Values for future orientation of Delay Discounting; CES = Participant self-reported emotional symptoms, CCP = Participant self-reported conduct problems, CPR = Participant self-reported peer relationship problems, CADHD = Participant self-reported ADHD symptoms; PES = Parent-reported emotional symptoms, PCP = Parent-reported conduct problems, PPR = Parent-reported peer relationship problems, PADHD = Parent-reported ADHD symptoms

**Table 2:** Bivariate Correlations among Variables

	kcertain	ktime	CES	CPR	CCP	CADHD	PES	PPR	PCP	PADHD
kcertain										
ktime	− 0.076^**^									
CES	0.045^**^	0.044^**^								
CPR	0.077^**^	− 0.015	0.376^**^							
CCP	− 0.033^**^	− 0.055^**^	0.212^**^	0.246^**^						
CADHD	0.001	− 0.018	0.325^**^	0.212^**^	0.454^**^					
PES	0.056^**^	0.007	0.429^**^	0.289^**^	0.151^**^	0.174^**^				
PPR	0.082^**^	− 0.032^*^	0.195^**^	0.435^**^	0.138^**^	0.112^**^	0.490^**^			
PCP	0.010	− 0.075^**^	0.060^**^	0.147^**^	0.384^**^	0.220^**^	0.355^**^	0.322^**^		
PADHD	0.039^**^	− 0.097^**^	0.075^**^	0.189^**^	0.337^**^	0.393^**^	0.393^**^	0.362^**^	0.548^**^	

Note: *p < .05, **p < .01; kcertain = Risk aversion; ktime = Future orientation; CES = Participant self-reported emotional symptoms, CCP = Participant self-reported conduct problems, CPR = Participant self-reported peer relationship problems, CADHD = Participant self-reported ADHD symptoms; PES = Parent-reported emotional symptoms, PCP = Parent-reported conduct problems, PPR = Parent-reported peer relationship problems, PADHD = Parent-reported ADHD symptoms

**Table 3:** Paired-sample t-test between Participant Self-reported and Parent-reported SDQ

	Mean	Std. Deviation	Std. Error Mean	t	df	Sig. (2-tailed)
CES - PES	0.301	0.502	0.006	50.116	6996	0.000
CPR - PPR	0.083	0.370	0.004	18.796	7010	0.000
CCP - PCP	0.114	0.325	0.004	29.312	7025	0.000
CADHD - PADHD	0.316	0.500	0.006	53.081	7063	0.000

CES = Participant self-reported emotional symptoms, CCP = Participant self-reported conduct problems, CPR = Participant self-reported peer relationship problems, CADHD = Participant self-reported ADHD symptoms; PES = Parent-reported emotional symptoms, PCP = Parent-reported conduct problems, PPR = Parent-reported peer relationship problems, PADHD = Parent-reported ADHD symptoms

**3.1 Hypothesis****1**: **Parent-reported adolescent mental health problems can be captured by a general factor using a bi-factor modelling approach**.

**Fig. 1:**
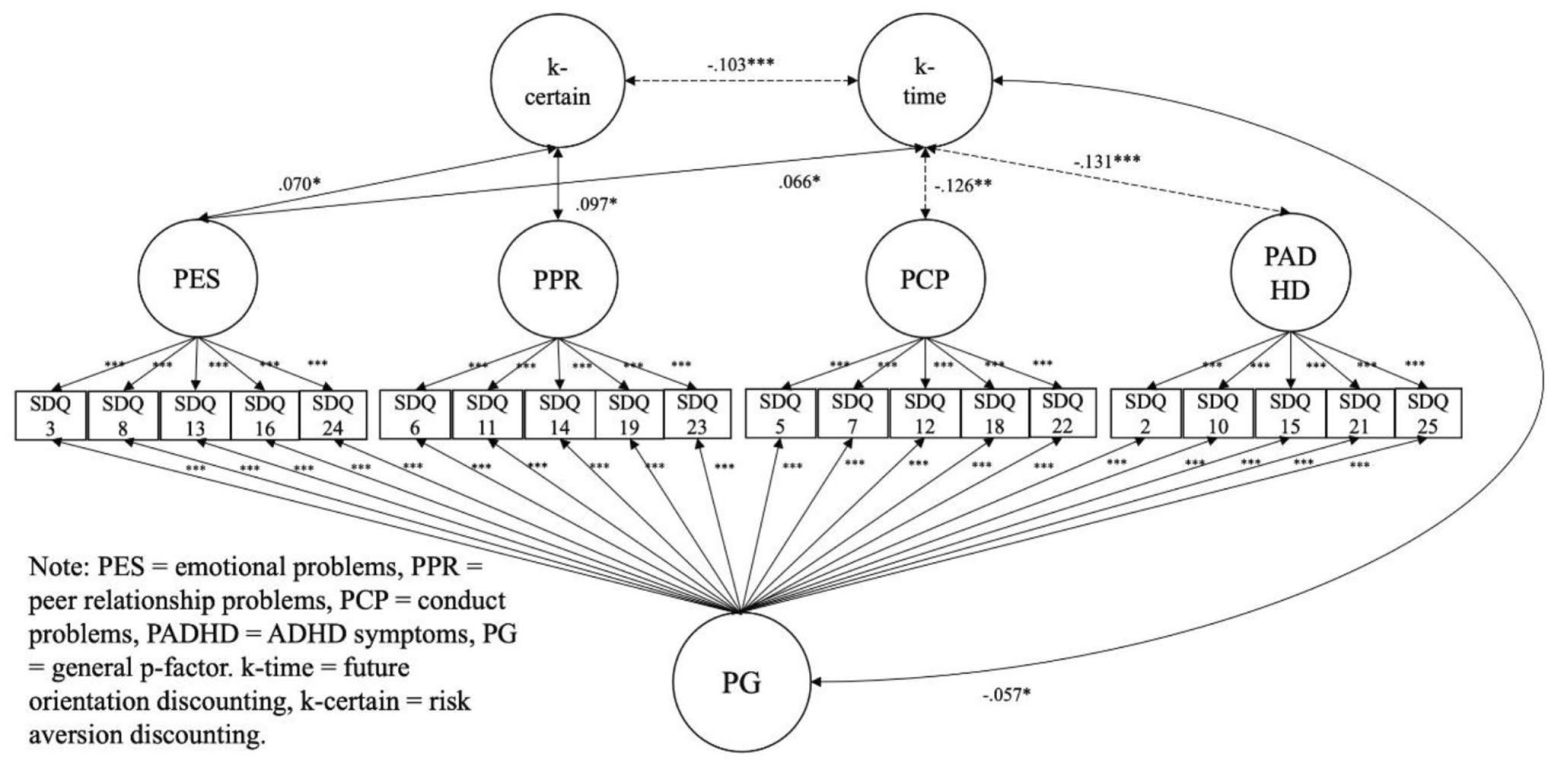
Bi-factor Analysis with Parent Reported SDQ

**Parent-reported SDQ (**Fig. 1, **Tables S4 & S6)**: Results suggested that our first hypothesis, that parent-reported adolescent mental health problems could be captured by a general factor using a bi-factor modelling approach was supported. The model fits for the bi-factor correlation analysis using the parent-reported SDQ were CFI = 0.919, TLI = 0.896, RMSEA = 0.057, SRMR = 0.062, which mostly met the conventional standards for good fit (CFI and TLI > 0.90; RMSEA and SRMR < 0.08). All 20 SDQ symptom items significantly loaded on a general factor (*p*s < 0.001), as well as on each of the theoretical subfactors (*p*s < 0.001), with only 3 items loadings < |0.30| on subfactors.

**3.2 Hypothesis****2**: **Adolescent self-reported mental health problems can be captured by a general factor using a bi-factor modelling approach**.

### Adolescent self-reported SDQ (Fig. 2, Tables S5 & S7)

Our second hypothesis, that adolescent self-reported mental health problems could be captured with a general factor using a bi-factor modelling approach was only partially supported. The model fits for the bi-factor correlation analysis using the adolescent self-reports were CFI = 0.882, TLI = 0.849, RMSEA = 0.064, SRMR = 0.065, which inadequately met the conventional standards only with RMSEA and SRMR for good fit (CFI and TLI > 0.90; RMSEA and SRMR < 0.08). However, all 20 SDQ symptom items significantly loaded on a general factor (*p*s < 0.001), as well as on each of the theoretical subfactors (*p*s < 0.001), with the loadings of 2 items on p-factor and 3 items on subfactors < |0.30|.

**Fig. 2:**
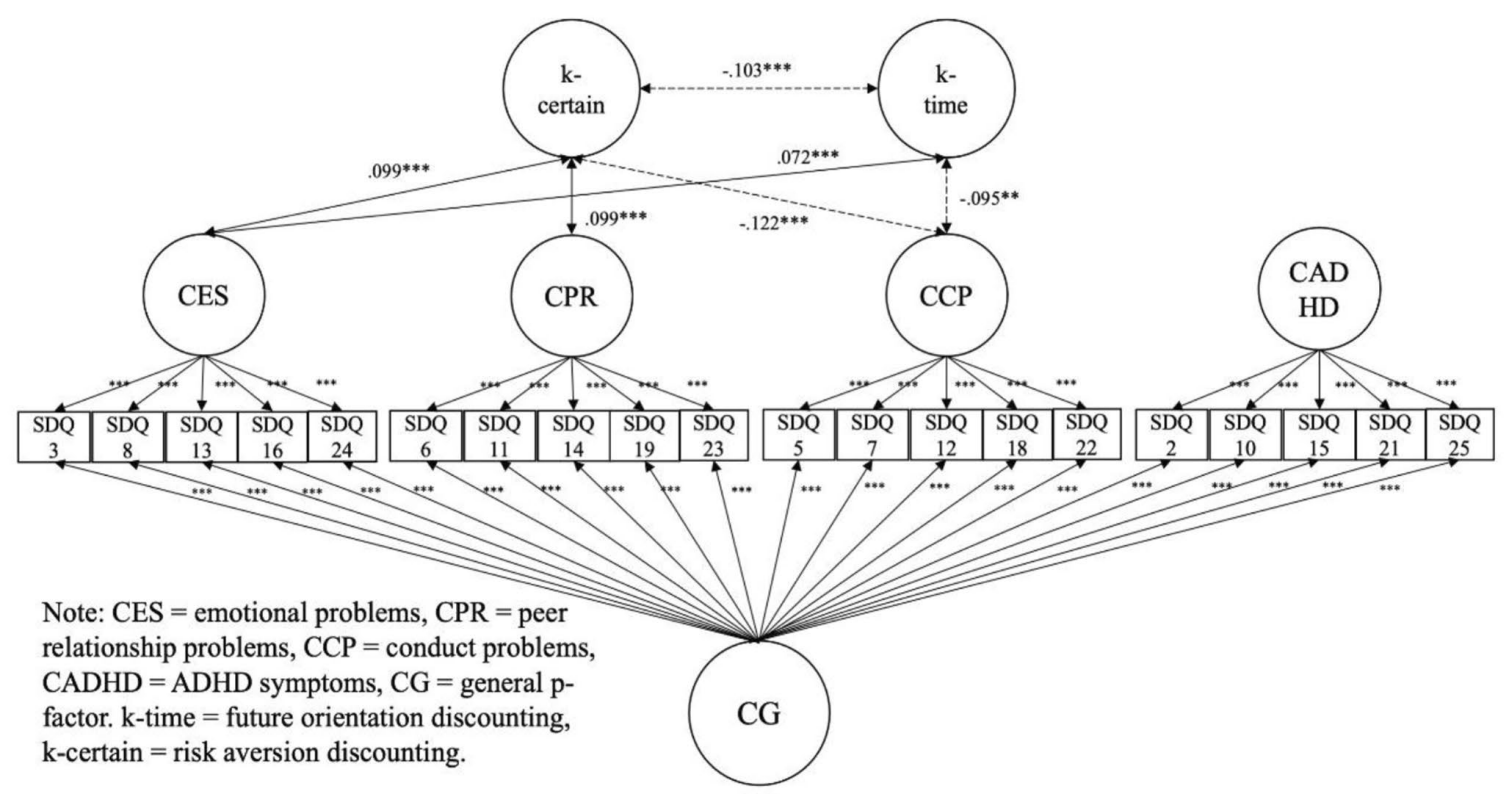
Bi-factor Analysis with Self-reported SDQ

**3.3 Hypothesis****3**: **The future orientation component of delay discounting will transdiagnostically predict the p-factor operationalised in these bi-factor models**.

**Parent-reported SDQ (**Fig. 1, **Tables S4 & S6)**: Our third hypothesis, that the future orientation component of delay discounting would transdiagnostically predict the p-factor operationalised within a bi-factor model was only supported based on the parent-reported data. Results of correlating these factors with the delay discounting suggested that future orientation (the higher k value, the longer time delayed) was negatively associated with the general p-factor (*r* = − .057, *p* < .05; the higher score, the higher mental issues), conduct problems (*r* = − .126, *p* < .05) and ADHD (*r* = − .131, *p* < .001); however, future orientation was positively associated with emotion problems (*r* = .066, *p* < .05). Risk aversion (the higher k value, the higher risk aversion or certainty preference) was not associated with the general p-factor (*r* = − .012, p = .677), but was positively associated with emotion problems (*r* = .070, *p* < .05) and peer problems (*r* = .097, *p < .*05). In this model, future orientation and risk aversion were negatively associated (*r* = − .103, *p < .*001).

### Adolescent self-reported SDQ (Fig. 2, Tables S5 & S7)

The bi-factor correlation results suggested that future orientation was not associated with the general p-factor (*r* = − .012, *p* = .579), but was positively associated with emotional problems (*r* = .072, *p* < .001) and negatively associated with conduct problems (*r* = − .095, *p* < .01). Risk aversion was not associated with the general p-factor (*r* = .008, *p* = .711), but positively associated with emotional problems (*r* = .099, *p* < .001) and peer problems (*r* = .099, *p < .*001), while negatively associated with conduct problems (*r* = − .122, *p* < .001). Future orientation and risk aversion were also negatively associated in this model (*r* = − .103, *p < .*001).

### Additional and Exploratory Analyses

#### Multiple Group Confirmatory Factor Analysis (MG-CFA)

As shown in Tables S8-S10 in supplementary materials, metric invariance was supported by models (ΔCFI ≥ − 0.01, ΔRMSEA ≤ 0.015, ΔSRMR ≤ 0.03; Chen, 2007) for all the CFA, bi-factor, and bi-factor SEM models across informants. In addition, the standards for metric invariance for categorical data also held as ΔRMSEA = − 0.004; Δχ^2^ = 609.54, Δdf = 35, *p* < .001; ΔCFI = 0.004 (Rutkowski & Svetina, 2017). These MG-CFA results empirically supported meaningful descriptive comparisons and interpretations of both parent-reposted and self-reported results from the two separate bi-factor models in Figs. 1 and 2.

#### SEM Analysis for each Subfactor

**Fig. 3:**
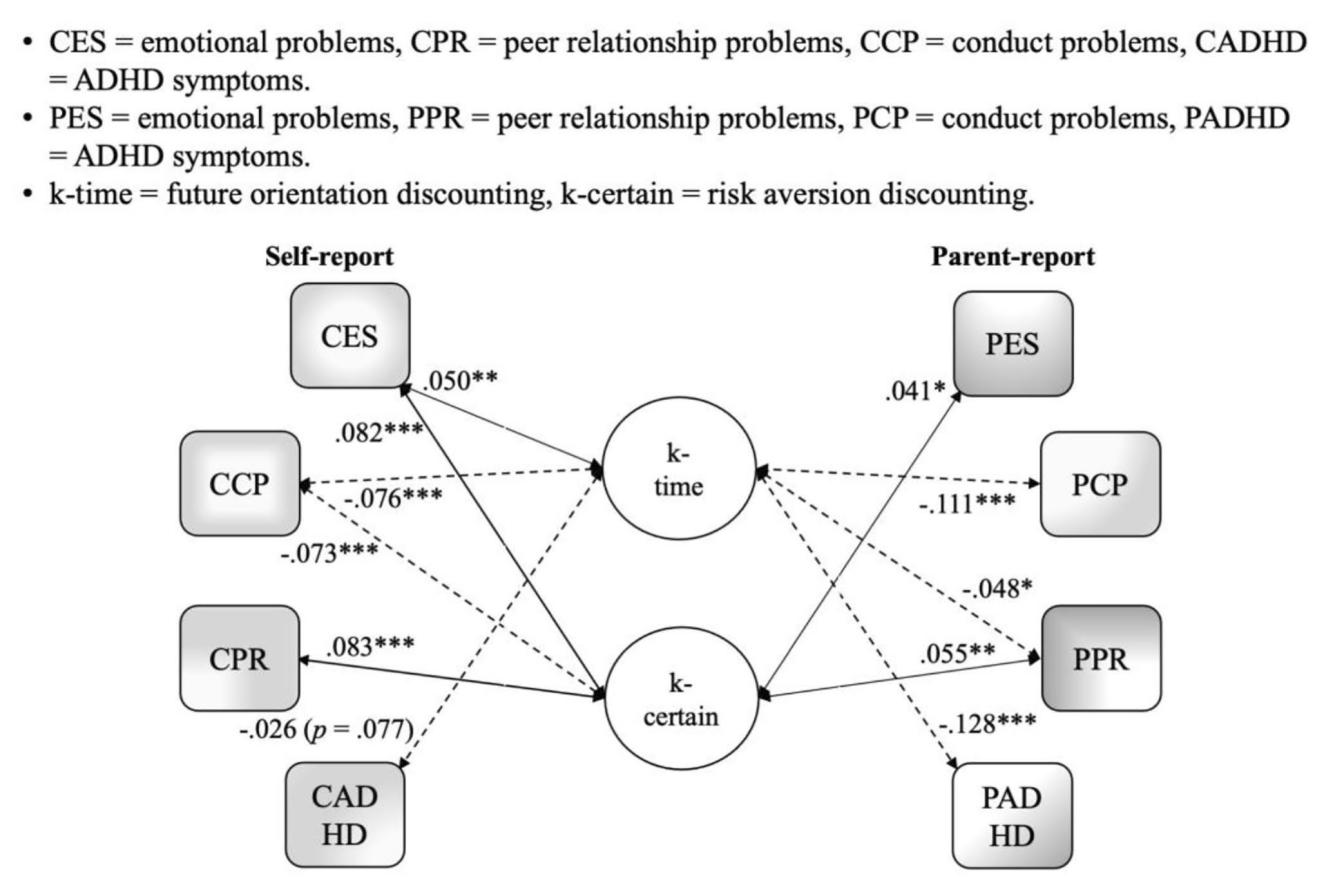
Subfactor SEMs using Parent Reported SDQ and Self-reported SDQ Note: Fig. 3 illustrates the results of 8 different models, one each for each mental health sub-factor and informant

The respective associations of both future orientation and risk aversion with each of the SDQ mental symptom variables were also explored using separate structural equation models (SEMs) for each adolescent self-reported and parent-reported SDQ subscale, resulting in 8 separate subfactor SEMs. The correlation results from these separate models are illustrated together in Fig. 3 to facilitate a descriptive comparison of results from both informants in a divergent approach, and to further substantiate and clarify the transdiagnostic versus domain-specific nature of both future orientation and risk aversion (further detailed explanations are found in supplementary materials page 11 with Tables S11-S14).

## Discussion

The purpose of the current study was to examine whether different elements (future orientation vs. risk/reward seeking) of delay discounting are associated transdiagnostically and domain-specifically with adolescent mental health problems. Using data from the Millennium Cohort Study age 17 sweep, we fit bi-factor models to parent- and self-reported data on ADHD symptoms, emotional problems, conduct problems, and peer problems. Given that metric measurement invariance across parent and youth self-reports was established by the multiple group confirmatory factor analyses, we could also descriptively compare the results across informants in a divergent approach. This also allowed us to see which associations replicated across both informants, suggesting that these associations may be more robust. We found that future orientation was associated with the general p-factor in a bi-factor model using parent- but not adolescent self-reported SDQ; however, risk aversion was consistently not associated with the p-factor based on either informant’s reports. Within the parent- and self-report bi-factor models, consistently, above the association with p-factor, future orientation was negatively associated with the specific factors of conduct problems and ADHD symptoms but positively associated with emotional problems, while risk aversion was negatively associated with conduct problems but positively associated with emotional and peer problems. Parents and adolescents showed report variations regarding: risk aversion with conduct problems association, future orientation with ADHD association, and future orientation with general psychopathology, as shown in Figs. 1 and 2.

These findings unanimously support making a distinction between the competing future orientation and risk reward-seeking process of delay discounting (e.g., Jiang & Dai, 2021; Steinberg et al., 2009), proposed to be underpinned by two brain systems results: the limbic structure associated with immediate rewards/sensation seeking, and the lateral prefrontal cortex and posterior parietal cortex associated with future orientation and self-regulation (McClure et al., 2004). Indeed, in the current study, these components consistently showed differential associations with general and specific mental health dimensions across both informants. Only the future orientation element of delay discounting received support from an association with general psychopathology (albeit limited to a single informant, parents) suggesting that its effects are better justified as transdiagnostic on general psychopathology than the risk element. In fact, research has suggested that the age differences in delay discounting performance during adolescence are significantly mediated by future orientation but not impulsivity (Steinberg et al., 2009). Future time is indeed perceived subjectively, which directly influences the degree of future orientation preference during decision-making (Leboeuf, 2006; Read et al., 2005), and importantly, is malleable (Jiang & Dai, 2021). Given the prevalence of co-occurrence of psychiatric disorders (Caspi & Moffitt, 2018), developing techniques promoting future orientation likely represents a promising target for efficient interventions that can impact a range of symptom domains at once or promote general resilience. For example, training adolescents’ future orientation based on its dimensions (e.g., vividness, positivity, connectedness; see reviews by Kooij et al., 2018; Sedikides et al., 2023) and promoting their generalized use in daily life could help improve their self-regulative function and adaptation (e.g., goal setting/monitoring/operating, Eisen et al., 2003; Griffin et al., 2009; Baird et al., 2021; decision-making, emotion regulation, prospective memory, and spatial navigation, Schacter et al., 2017). This is also substantiated by a meta-analysis on successful manipulations using future-oriented intervention techniques to improve delay discounting performance (Rung & Madden, 2018; Ye et al., 2022). Some well-established therapies also include future orientation as an element (e.g., commit present actions to value-oriented future goals from Acceptance and Commitment Therapy, A-Tjak et al., 2015; challenge negative thoughts of self, present life, and future from Cognitive Behavioural Therapy, Beck & Emery, 1979). However, given only moderate associations based on a large sample was found in our study, further research evidence is necessary to investigate its promotive effect on general pathology.

Although only future orientation appeared to show a transdiagnostic relation to mental health problems, both future orientation and risk/reward elements were associated with specific dimensions of mental health problems over and above general mental health problems variance captured by the p-factor. Notably, these were not universally acting as promotive/risk factors but varied in the direction of their relations to different domains of mental health problems. Specifically, while greater future orientation (higher k value) was related to lower ADHD (parent reports) and conduct problem symptoms (both informants), it was consistently related to higher emotional problems (both informants). This suggests while future orientation may help adolescents to engage in behaviour characterised by self-control (Baird et al., 2021), where this is excessive it may result in negative effects, perhaps partially due to a failure to appreciate the present. Indeed, it has previously been found that more balanced time perspectives that neither over-weigh the present nor the future are optimal for good mental health (Stolarski et al., 2020). Interventions aims a good balance between temporal perspectives would be suggested, and/or training adolescents with emotion regulation skills in addition to future time perspective.

It is also notable in Fig. 3 illustrating subfactor SEMs that only the future orientation (not risk aversion) element of delay discounting was consisitently and negatively associated with ADHD symptoms across informants. This further clarifies a specific future orientation pathway associated with ADHD compared to previous studies (Patros et al., 2016). This future orientation deficit is also in line with the well-established time perception deficit of ADHD (e.g., shorter time duration judgement; see reviews by Mette, 2023; Nejati & Yazdani, 2020; Ptacek et al., 2019), indicating the discrimination between temporal orientation and risk/reward-seeking is important because related intervention strategies differ.

Similarly, higher risk/reward-seeking (lower risk aversion) was consistently associated with lower peer and emotion problems (both informants) but associated with higher conduct problems (self-reports). This suggests that while excessive risk-taking may be a marker for externalising problems, internalising problems seem associated with risk aversion or excessive certainty preference. The positive relation between risk aversion and peer problems is notable in the context of the magnified importance of peers in this stage of development (Steinberg et al., 2008; Icenogle & Cauffman, 2021). Indeed, in this stage of development, much risk-taking takes place in the context of peers and may be an important social activity that more risk-averse adolescents may miss out on. Further, actively making friends itself could be “risky” since there is uncertainty surrounding social rejection or failure. Therefore, “adventurous” adolescents could initiate more peer interactions, as well as behave to better conform to their risk-taking sub-culture (e.g., peer presence increased adolescents’ risk-taking behaviours, Icenogle & Cauffman, 2021), and therefore less likely to have peer problems. Overall, analogous to the future orientation element of delay discounting, this supports the idea that moderate levels of risk-taking (or certainty preference) may be optimal for mental health. Indeed, there is a well-established normative increase in risk-taking in adolescence (Duell et al., 2018) and it has been argued by some degree of risk-taking may be an important part of this phase of development (e.g., Icenogle & Cauffman, 2021; Romer et al., 2017). As such, interventions focused on reducing adolescent risk-taking would be suggested to not necessarily aim to eliminate risk-taking behaviours but to teach optimal levels and/or to channel excessive risk-taking tendencies into “positive” forms of risk-taking, such as sports competition (e.g., Duell & Steinberg, 2019).

### Limitations and Future Directions

As has been noted in previous research, model selection based on fit statistics between the bi-factor model and other measurement models may not be adequate to indicate the better fitting model, and the choice measurement model must therefore be based on background knowledge and/or the purpose of the analysis. We selected a bi-factor model as a means to separate general and specific factor variance; however, it is not necessarily a representation of the true underlying structure of psychopathology. In addition, only one sweep of MCS has examined delay discounting tasks so far, therefore, the present study can only provide evidence of cross-sectional associations. Future studies with more than one sweep of data available could employ longitudinal analysis methods to further clarify the within-person level associations between these concepts and their potential changes over development. Addressing possible confounding processes in the associations found in the present study could also be further explored through experimental or counterfactual designs. Subgroup differences (e.g., by gender) could also be explored in future research.

## Conclusion

In this study, specific associations were delineated by distinguishing the future orientation and risk/reward-seeking elements of delay discounting. Future orientation played a promotive role with respect to conduct problems and ADHD symptoms and a risk role with respect to emotional problems. Risk-taking played a risk role with respect to conduct problems and a promotive role with respect to emotional and peer problems.

### Electronic Supplementary Material

Below is the link to the electronic supplementary material.

Supplementary Material 1
