# Supplementary material for: Associations Between Reward and Future-Related Orientations and General and Specific Mental Health Issues in Adolescence
Source: Res Child Adolesc Psychopathol. 2023 Oct 7;52(3):385–97. doi: 10.1007/s10802-023-01136-y (PMC10896876; doi:10.1007/s10802-023-01136-y)
Supplement: Supplementary file 1 — Supplementary Material 1 [file 10802_2023_1136_MOESM1_ESM.docx]

**Supporting Information**

**Figure S1. Future Orientation Discounting Task (Future Preference)**

**
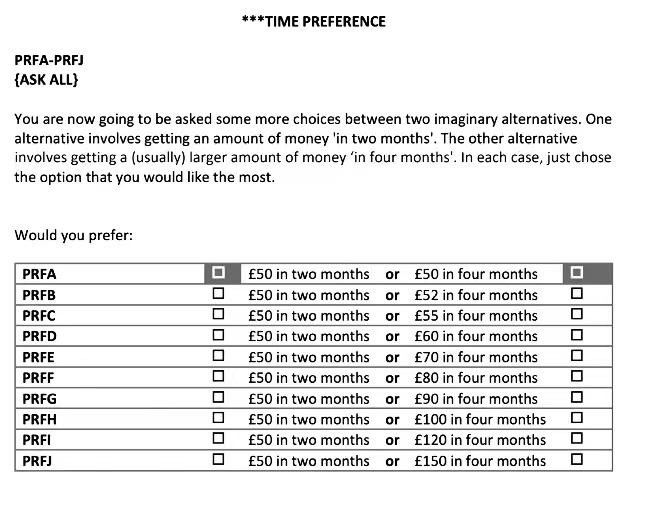
**

**Figure S2. Risk Aversion Discounting Task (Certainty Preference)**

**
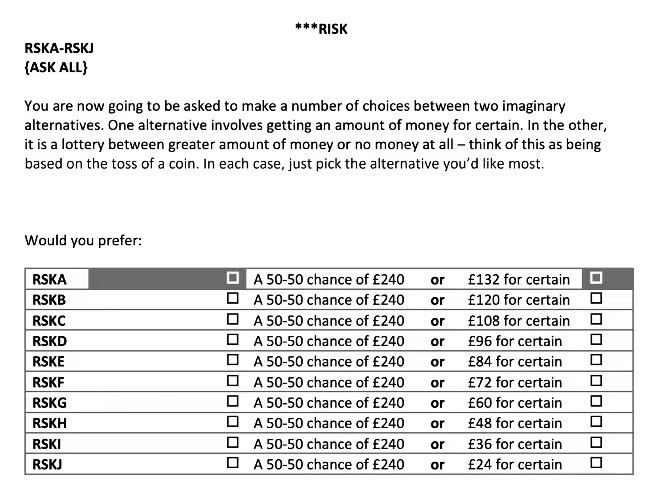
**

| **Table S1. k Values for Future Orientation Discounting Task** | | | | | |  |
| --- | --- | --- | --- | --- | --- | --- |
| **Index** | **Money (A=£50)** | **ED50 (£) if last choice is(kED50=1):** | | **k(k↓=time↓) if last choice is:** | |  |
|  |  | In 2 Months | In 4 Months | In 2 Months | In 4 Months |  |
| DD11 | 50 | 50.990195 | 50 | .0196116 | .02 |  |
| DD12 | 52 | 53.478968 | 50.990195 | .0186989 | .0196116 |  |
| DD13 | 55 | 57.445626 | 53.478968 | .0174078 | .0186989 |  |
| DD14 | 60 | 64.807407 | 57.445626 | .0154303 | .0174078 |  |
| DD15 | 70 | 74.833148 | 64.807407 | .0133631 | .0154303 |  |
| DD16 | 80 | 84.852814 | 74.833148 | .0117851 | .0133631 |  |
| DD17 | 90 | 94.86833 | 84.852814 | .0105409 | .0117851 |  |
| DD18 | 100 | 109.54451 | 94.86833 | .0091287 | .0105409 |  |
| DD19 | 120 | 134.16408 | 109.54451 | .0074536 | .0091287 |  |
| DD20 | 150 | 150 | 134.16408 | .0066667 | .0074536 |  |
| 1=£50 in 2 months, 2=in 4 months; the first choice of 2 is the indifferent point; (£)↑=delay preference↓ | | | | | |  |
|  |  |  |  |  |  |  |
| Trial↑ (£)↑= k value↓, 1=delay preference↓: **k value**↑**=delay preference**↑ (future orientation↑) | | | | | |  |
|  |  |  |  |  |  |  |

| **Table S2. k Values for Risk Aversion (Certainty Preference) Discounting Task** | | | | | |  |
| --- | --- | --- | --- | --- | --- | --- |
| **Index** | **Money (A=240£)** | **ED50 (£) if last choice is(kED50=1):** | | **k(k↑=** **Risk↓) if last choice is:** | |  |
|  |  | 50% chance | 100% chance | 50% chance | 100% chance |  |
| DD1 | 132 | 132 | 125.85706 | .0075758 | .0079455 |  |
| DD2 | 120 | 125.85706 | 113.842 | .0079455 | .0087841 |  |
| DD3 | 108 | 113.842 | 101.82338 | .0087841 | .0098209 |  |
| DD4 | 96 | 101.82338 | 89.799777 | .0098209 | .0111359 |  |
| DD5 | 84 | 89.799777 | 77.768888 | .0111359 | .0128586 |  |
| DD6 | 72 | 77.768888 | 65.726707 | .0128586 | .0152145 |  |
| DD7 | 60 | 65.726707 | 53.665631 | .0152145 | .0186339 |  |
| DD8 | 48 | 53.665631 | 41.569219 | .0186339 | .0240563 |  |
| DD9 | 36 | 41.569219 | 29.393877 | .0240563 | .0340207 |  |
| DD10 | 24 | 29.393877 | 24 | .0340207 | .0416667 |  |
| 1=50%-50% chance of £240, 2=for certain; the first choice of 1 is the indifferent point; (£)↓= certainty preference↑ (risk taking↓) | | | | | |  |
|  |  |  |  |  |  |  |
| Trial↑ (£)↓= k value↑, 1= certainty preference↑: **k value↑= certainty preference↑ (risk taking↓ risk aversion↑)** | | | | | |  |
|  |  |  |  |  |  |  |

| **Table S3. SDQ Bifactor Correlation Model Fits** | | | | |
| --- | --- | --- | --- | --- |
| Model | CFI | TLI | RMSEA | SRMR |
| PSDQ Bifactor Correlation Model (N=9323) | .919 | .896 | .057 | .062 |
| CSDQ Bifactor Correlation Model (N=9881) | .882 | .849 | .064 | .065 |
| CSDQ= Child Self-report SDQ scores; PSDQ= Parents Report SDQ scores; CFI=Robust Comparative Fit Index; TLI=Robust Tucker-Lewis Index. | | | | |

| **Table S4. Parents Report SDQ Bifactor Model Factor Loadings (N=9323)** | | | | | | |
| --- | --- | --- | --- | --- | --- | --- |
| Latent Variables | Loading | *p*(>\|z\|) |  | Latent Variable | Loading | *p*(>\|z\|) |
| PG =~ |  |  |  | PES =~ |  |  |
| PSDQ2 | .555 | .000 |  | PSDQ3 | .366 | .000 |
| PSDQ3 | .389 | .000 |  | PSDQ8 | .652 | .000 |
| PSDQ5 | .682 | .000 |  | PSDQ13 | .486 | .000 |
| PSDQ6 | .493 | .000 |  | PSDQ16 | .449 | .000 |
| RPSDQ7 | .524 | .000 |  | PSDQ24 | .596 | .000 |
| PSDQ8 | .531 | .000 |  | PPR =~ |  |  |
| PSDQ10 | .646 | .000 |  | PSDQ6 | .369 | .000 |
| RPSDQ11 | .512 | .000 |  | RPSDQ11 | .511 | .000 |
| PSDQ12 | .673 | .000 |  | RPSDQ14 | .495 | .000 |
| PSDQ13 | .625 | .000 |  | PSDQ19 | .290 | .000 |
| RPSDQ14 | .597 | .000 |  | PSDQ23 | .349 | .000 |
| PSDQ15 | .643 | .000 |  | PCP =~ |  |  |
| PSDQ16 | .598 | .000 |  | PSDQ5 | .196 | .000 |
| PSDQ18 | .636 | .000 |  | RPSDQ7 | .319 | .000 |
| PSDQ19 | .596 | .000 |  | PSDQ12 | .284 | .000 |
| RPSDQ21 | .545 | .000 |  | PSDQ18 | .569 | .000 |
| PSDQ22 | .610 | .000 |  | PSDQ22 | .589 | .000 |
| PSDQ23 | .360 | .000 |  | PADHD =~ |  |  |
| PSDQ24 | .577 | .000 |  | PSDQ2 | .529 | .000 |
| RPSDQ25 | .615 | .000 |  | PSDQ10 | .528 | .000 |
|  |  |  |  | PSDQ15 | .509 | .000 |
|  |  |  |  | RPSDQ21 | .330 | .000 |
|  |  |  |  | RPSDQ25 | .473 | .000 |
| PES = Parent report emotional symptoms, PCP = Parent report conduct problems, PPR = Parent report peer relationship problems, PADHD = Parent report ADHD symptoms, PG = general factor from parent report SDQ. | | | | | | |

| **Table S5. Child Self-report SDQ Bifactor Model Factor Loadings (N=9881)** | | | | | | |
| --- | --- | --- | --- | --- | --- | --- |
| Latent Variables | Loading | *p*(>\|z\|) |  | Latent Variable | Loading | *p*(>\|z\|) |
| CG =~ |  |  |  | CES =~ |  |  |
| CSDQ2 | .436 | .000 |  | CSDQ3 | .276 | .000 |
| CSDQ3 | .476 | .000 |  | CSDQ8 | .739 | .000 |
| CSDQ5 | .625 | .000 |  | CSDQ13 | .468 | .000 |
| CSDQ6 | .431 | .000 |  | CSDQ16 | .539 | .000 |
| RCSDQ7 | .297 | .000 |  | CSDQ24 | .632 | .000 |
| CSDQ8 | .400 | .000 |  | CPR =~ |  |  |
| CSDQ10 | .565 | .000 |  | CSDQ6 | .376 | .000 |
| RCSDQ11 | .283 | .000 |  | RCSDQ11 | .541 | .000 |
| CSDQ12 | .392 | .000 |  | RCSDQ14 | .528 | .000 |
| CSDQ13 | .608 | .000 |  | CSDQ19 | .374 | .000 |
| RCSDQ14 | .388 | .000 |  | CSDQ23 | .377 | .000 |
| CSDQ15 | .584 | .000 |  | CCP =~ |  |  |
| CSDQ16 | .407 | .000 |  | CSDQ5 | .251 | .000 |
| CSDQ18 | .519 | .000 |  | RCSDQ7 | .367 | .000 |
| CSDQ19 | .540 | .000 |  | CSDQ12 | .629 | .000 |
| RCSDQ21 | .393 | .000 |  | CSDQ18 | .443 | .000 |
| CSDQ22 | .385 | .000 |  | CSDQ22 | .434 | .000 |
| CSDQ23 | .267 | .000 |  | CADHD =~ |  |  |
| CSDQ24 | .389 | .000 |  | CSDQ2 | .623 | .000 |
| RCSDQ25 | .444 | .000 |  | CSDQ10 | .556 | .000 |
|  |  |  |  | CSDQ15 | .486 | .000 |
|  |  |  |  | RCSDQ21 | .272 | .000 |
|  |  |  |  | RCSDQ25 | .403 | .000 |
| CES = Participant self-report emotional symptoms, CCP = Participant self-report conduct problems, CPR = Participant self-report peer relationship problems, CADHD = Participant self-report ADHD symptoms, CG = general factor from participant self-report SDQ. | | | | | | |

| **Table S6. Parents Report SDQ Bifactor SEM Correlations (N=9323)** | | | | | | | | | | | | |
| --- | --- | --- | --- | --- | --- | --- | --- | --- | --- | --- | --- | --- |
|  | *r* | | *p*(>\|z\|) | |  | |  | | *r* | | *p*(>\|z\|) | |
| ktime~~PG | -.057* | | .040 | |  | | kcertain~~PG | | -.012 | | .677 | |
|  |  | |  | |  | |  | |  | |  | |
| ktime~~PES | .066* | | .031 | |  | | kcertain~~PES | | .070* | | .023 | |
| ktime~~PCP | -.126** | | .006 | |  | | kcertain~~PCP | | .003 | | .956 | |
| ktime~~PPR | -.006 | | .871 | |  | | kcertain~~PPR | | .097* | | .014 | |
| ktime~~PADHD | -.131*** | | .000 | |  | | kcertain~~PADHD | | -.007 | | .859 | |
|  | |  | |  | |  | |  | |  | |  |
| ktime~~kcertain | | -.103*** | | .000 | |  | |  | |  | |  |
| PES = Parent report emotional symptoms, PCP = Parent report conduct problems, PPR = Parent report peer relationship problems, PADHD = Parent report ADHD symptoms, PG = general factor from parent report SDQ. ktime = future orientation discounting, kcertain = risk aversion discounting (certainty preference). | | | | | | | | | | | | |

| **Table S7. Child Self-report SDQ Bifactor SEM Correlations (N=9881)** | | | | | | |
| --- | --- | --- | --- | --- | --- | --- |
|  | *r* | *p*(>\|z\|) |  |  | *r* | *p*(>\|z\|) |
| ktime~~CG | -.012 | .579 |  | kcertain~~CG | .008 | .711 |
|  |  |  |  |  |  |  |
| ktime~~CES | .072*** | .000 |  | kcertain~~CES | .099*** | .000 |
| ktime~~CCP | -.095** | .001 |  | kcertain~~CCP | -.122*** | .000 |
| ktime~~CPR | -.019 | .432 |  | kcertain~~CPR | .099*** | .000 |
| ktime~~CADHD | -.022 | .352 |  | kcertain~~CADHD | -.005 | .829 |
|  |  |  |  |  |  |  |
| ktime~~kcertain | -.103*** | .000 |  |  |  |  |
| CES = Participant self-report emotional symptoms, CCP = Participant self-report conduct problems, CPR = Participant self-report peer relationship problems, CADHD = Participant self-report ADHD symptoms, CG = general factor from participant self-report SDQ. ktime = future orientation discounting, kcertain = risk aversion discounting (certainty preference). | | | | | | |

| **Table S8. Multiple Group Confirmatory Factor Analysis** | | | | | | | | | | |
| --- | --- | --- | --- | --- | --- | --- | --- | --- | --- | --- |
|  | chi-square (χ2) | df | CFI | TLI | RMSEA | SRMR | ΔCFI | ΔRMSEA | ΔSRMR | Invariance? |
| All report | 10180.731 | 164.000 | 0.929 | 0.918 | 0.059 | 0.060 | NA | NA | NA | NA |
| Parent report | 4145.845 | 190.000 | 0.938 | 0.929 | 0.056 | 0.060 | NA | NA | NA | NA |
| Child report | 6363.428 | 164.000 | 0.897 | 0.881 | 0.062 | 0.068 | NA | NA | NA | NA |
| Configural | 10607.130 | 328.000 | 0.918 | 0.905 | 0.060 | 0.065 | NA | NA | NA | NA |
| **Metric** | **10386.751** | **344.000** | **0.919** | **0.911** | **0.058** | **0.068** | **0.001** | **-0.002** | **0.003** | **Yes** |
| Scalar | 14285.866 | 360.000 | 0.888 | 0.882 | 0.066 | 0.066 | -0.031 | 0.008 | -0.002 | No |
| Strict | 14285.866 | 360.000 | 0.888 | 0.882 | 0.066 | 0.066 | 0.000 | 0.000 | 0.000 | NA |

Cut-offs for Scalar/Strict invariance: ΔCFI ≥ -.01, ΔRMSEA ≤ .015, ΔSRMR ≤ .03; cut-offs for Scalar/Strict invariance: ΔCFI ≥ -.01, ΔRMSEA ≤ .015, ΔSRMR ≤ .01 (Chen, 2007). All-report=combined both parent and youth reported data.

| **Table S9. Multiple Group Bi-factor Analysis** | | | | | | | | | | |
| --- | --- | --- | --- | --- | --- | --- | --- | --- | --- | --- |
|  | chi-square (χ2) | df | CFI | TLI | RMSEA | SRMR | ΔCFI | ΔRMSEA | ΔSRMR | Invariance? |
| All report | 12473.718 | 150.000 | 0.913 | 0.890 | 0.068 | 0.067 | NA | NA | NA | NA |
| Parent report | 5528.157 | 150.000 | 0.917 | 0.895 | 0.068 | 0.067 | NA | NA | NA | NA |
| Child report | 7311.347 | 150.000 | 0.881 | 0.850 | 0.070 | 0.071 | NA | NA | NA | NA |
| Configural | 12902.935 | 300.000 | 0.899 | 0.872 | 0.069 | 0.070 | NA | NA | NA | NA |
| **Metric** | **12210.264** | **335.000** | **0.905** | **0.892** | **0.064** | **0.075** | **0.006** | **-0.005** | **0.005** | **Yes** |
| Scalar | 13839.722 | 350.000 | 0.892 | 0.883 | 0.066 | 0.074 | -0.013 | 0.002 | -0.001 | No |
| Strict | 13839.722 | 350.000 | 0.888 | 0.882 | 0.066 | 0.066 | -0.004 | 0.000 | -0.008 | NA |

Cut-offs for Scalar/Strict invariance: ΔCFI ≥ -.01, ΔRMSEA ≤ .015, ΔSRMR ≤ .03; cut-offs for Scalar/Strict invariance: ΔCFI ≥ -.01, ΔRMSEA ≤ .015, ΔSRMR ≤ .01 (Chen, 2007). All-report=combined both parent and youth reported data.

| **Table S10. Multiple Group Bi-factor SEM Analysis** | | | | | | | | | | |
| --- | --- | --- | --- | --- | --- | --- | --- | --- | --- | --- |
|  | chi-square (χ2) | df | CFI | TLI | RMSEA | SRMR | ΔCFI | ΔRMSEA | ΔSRMR | Invariance? |
| All report | 12716.277 | 180.000 | 0.915 | 0.890 | 0.060 | 0.062 | NA | NA | NA | NA |
| Parent report | 5630.586 | 180.000 | 0.919 | 0.896 | 0.057 | 0.062 | NA | NA | NA | NA |
| Child report | 7477.711 | 180.000 | 0.882 | 0.849 | 0.064 | 0.065 | NA | NA | NA | NA |
| Configural | 13168.224 | 360.000 | 0.901 | 0.873 | 0.061 | 0.064 | NA | NA | NA | NA |
| **Metric** | **12703.270** | **395.000** | **0.905** | **0.889** | **0.057** | **0.069** | **0.004** | **-0.004** | **0.005** | **Yes** |
| Scalar | 13989.847 | 428.000 | 0.895 | 0.887 | 0.057 | 0.068 | -0.010 | 0.000 | -0.001 | Yes |
| Strict | 13989.847 | 428.000 | 0.895 | 0.887 | 0.057 | 0.068 | 0.000 | 0.000 | 0.000 | Yes |

Cut-offs for Scalar/Strict invariance: ΔCFI ≥ -.01, ΔRMSEA ≤ .015, ΔSRMR ≤ .03; cut-offs for Scalar/Strict invariance: ΔCFI ≥ -.01, ΔRMSEA ≤ .015, ΔSRMR ≤ .01 (Chen, 2007). All-report=combined both parent and youth reported data.

**SEM analysis Results for each subfactors**

The model fits for each SEMs are provided in Table S11 (child-reported) and Table S12 (parents reported), and all met the conventional standards for good fit (CFI and TLI >.90; RMSEA and SRMR <.08). The item loadings were also all significant (*p*s<.001), as shown in Table S13. The Correlation results are provided in Table S14. They suggest:

1) future orientation discounting was positively and significantly associated with child self-reported emotional problems (*r* = .050, *p* < .01) indicating the longer future time delay one preferred the more emotion problems on exhibited, but negatively and significantly associated with child self-reported conduct problems (*r* = -.076, *p* < .001), and parent-reported peer problems (*r* = -.048, *p* < .05), conduct problems (*r* = -.111, *p* < .001) and ADHD problems (*r* = -.128, *p* < .001), indicating time discounting was a protective factor against these problems; the negative association between time discounting and child self-reported ADHD was close to reach significance (*r* = -.026, *p* = .077);

2) risk aversion discounting was positively and significantly associated with child self-reported emotion problems (*r* = .082, *p* < .001) and peer problems (*r* = .083, *p* < .001), and parents reported emotional problems (*r* = .041, *p* < .05) and peer problems (*r* = .055, *p* < .01), indicating the less risk-taking and more certainty one preferred the more emotion and peer problems one showed, but negatively and significantly associated with child self-reported conduct problems (*r* = -.073, *p* < .001) indicating the more risk-taking the more conduct problems.

| **Table S11. CSDQ Subfactors SEM Model Fit** | | | | |
| --- | --- | --- | --- | --- |
| Model | CFI | TLI | RMSEA | SRMR |
| CES (N=9881) | .988 | .981 | .042 | .026 |
| CPR (N=9877) | .982 | .971 | .026 | .027 |
| CCP (N=9878) | .988 | .980 | .022 | .024 |
| CADHD (N=9879) | .935 | .895 | .100 | .057 |
| CFI=Robust Comparative Fit Index; TLI=Robust Tucker-Lewis Index; CES = Participant self-report emotional symptoms, CCP = Participant self-report conduct problems, CPR = Participant self-report peer relationship problems, CADHD = Participant self-report ADHD symptoms. | | | | |

| **Table S12. PSDQ Subfactors SEM Model Fit** | | | | |
| --- | --- | --- | --- | --- |
| Model | CFI | TLI | RMSEA | SRMR |
| PES (N=9323) | .985 | .975 | .049 | .030 |
| PPR (N=9323) | .988 | .980 | .025 | .025 |
| PCP (N=9323) | .987 | .978 | .028 | .037 |
| PADHD (N=9321) | .940 | .903 | .105 | .069 |
| CFI=Robust Comparative Fit Index; TLI=Robust Tucker-Lewis Index; PES = Parent report emotional symptoms, PCP = Parent report conduct problems, PPR = Parent report peer relationship problems, PADHD = Parent report ADHD symptoms. | | | | |

| **Table S13. Loadings of Single-factor SEMs with Time Discounting and Risk Discounting as Correlators** | | | | | | |
| --- | --- | --- | --- | --- | --- | --- |
| Participant self-report SDQ | | |  | Parent report SDQ | | |
| CES Model (N=9881) |  |  |  | PES Model (N=9323) |  |  |
| CES=~ | Loading | *p*(>\|z\|) |  | PES =~ | Loading | *p*(>\|z\|) |
| CSDQ3 | .510 | .000 |  | PSDQ3 | .534 | .000 |
| CSDQ8 | .826 | .000 |  | PSDQ8 | .831 | .000 |
| CSDQ13 | .730 | .000 |  | PSDQ13 | .780 | .000 |
| CSDQ16 | .674 | .000 |  | PSDQ16 | .738 | .000 |
| CSDQ24 | .739 | .000 |  | PSDQ24 | .837 | .000 |
| CPR Model (N=9877) |  |  |  | PPR Model (N=9323) |  |  |
| CPR=~ | Loading | *p*(>\|z\|) |  | PPR =~ | Loading | *p*(>\|z\|) |
| CSDQ6 | .564 | .000 |  | PSDQ6 | .612 | .000 |
| RCSDQ11 | .585 | .000 |  | RPSDQ11 | .716 | .000 |
| RCSDQ14 | .650 | .000 |  | RPSDQ14 | .778 | .000 |
| CSDQ19 | .631 | .000 |  | PSDQ19 | .639 | .000 |
| CSDQ23 | .471 | .000 |  | PSDQ23 | .505 | .000 |
| CCP Model (N=9878) |  |  |  | PCP Model (N=9323) |  |  |
| CCP =~ | Loading | *p*(>\|z\|) |  | PCP =~ | Loading | *p*(>\|z\|) |
| CSDQ5 | .588 | .000 |  | PSDQ5 | .676 | .000 |
| RCSDQ7 | .467 | .000 |  | RPSDQ7 | .635 | .000 |
| CSDQ12 | .723 | .000 |  | PSDQ12 | .729 | .000 |
| CSDQ18 | .690 | .000 |  | PSDQ18 | .826 | .000 |
| CSDQ22 | .576 | .000 |  | PSDQ22 | .825 | .000 |
| CADHD Model (N=9879) | |  |  | PADHD Model (N=9321) | |  |
| CADHD =~ | Loading | *p*(>\|z\|) |  | PADHD =~ | Loading | *p*(>\|z\|) |
| CSDQ2 | .737 | .000 |  | PSDQ2 | .759 | .000 |
| CSDQ10 | .774 | .000 |  | PSDQ10 | .821 | .000 |
| CSDQ15 | .764 | .000 |  | PSDQ15 | .821 | .000 |
| RCSDQ21 | .477 | .000 |  | RPSDQ21 | .634 | .000 |
| RCSDQ25 | .623 | .000 |  | RPSDQ25 | .789 | .000 |

Note: CES = Self-report emotional symptoms, CCP = Participant self-report conduct problems, CPR = Participant self-report peer relationship problems, CADHD = Participant self-report ADHD symptoms, CG = general factor from participant self-report SDQ. PES = Parent report emotional symptoms, PCP = Parent report conduct problems, PPR = Parent report peer relationship problems, PADHD = Parent report ADHD symptoms, PG = general factor from parent report SDQ.

| **Table S14. Correlations of Time Discounting and Risk Discounting with SDQ Factors in Single-factor SEMs** | | | | | | |
| --- | --- | --- | --- | --- | --- | --- |
| Participant self-report SDQ | | |  | Parent report SDQ | | |
| CES Model (N=9881) | *r* | *p*(>\|z\|) |  | PES Model (N=9323) | *r* | *p*(>\|z\|) |
| ktime ~~ CES | .050** | .001 |  | ktime ~~ PES | .004 | .810 |
| kcertain ~~ CES | .082*** | .000 |  | kcertain ~~ PES | .041* | .018 |
| CPR Model (N=9877) |  |  |  | PPR Model (N=9323) |  |  |
| ktime ~~ CPR | -.018 | .276 |  | ktime ~~ PPR | -.048* | .012 |
| kcertain ~~ CPR | .083*** | .000 |  | kcertain ~~ PPR | .055** | .004 |
| CCP Model (N=9878) |  |  |  | PCP Model (N=9323) |  |  |
| ktime ~~ CCP | -.076*** | .000 |  | ktime ~~ PCP | -.111*** | .000 |
| kcertain ~~ CCP | -.073*** | .000 |  | kcertain ~~ PCP | -.015 | .483 |
| CADHD Model (N=9879) |  |  |  | PADHD Model (N=9321) |  |  |
| ktime ~~ CADHD | -.026 | .077 |  | ktime ~~ PADHD | -.128*** | .000 |
| kcertain ~~ CADHD | .000 | .999 |  | kcertain ~~ PADHD | -.015 | .388 |
| Note: CES = Participant self-report emotional symptoms, CCP = Participant self-report conduct problems, CPR = Participant self-report peer relationship problems, CADHD = Participant self-report ADHD symptoms, CG = general factor from participant self-report SDQ. PES = Parent report emotional symptoms, PCP = Parent report conduct problems, PPR = Parent report peer relationship problems, PADHD = Parent report ADHD symptoms, PG = general factor from parent report SDQ. kcertain = Risk Aversion Discounting; ktime = Future Orientation Discounting. | | | | | | |
